# The effect of drug holidays on sexual dysfunction in men treated with selective serotonin reuptake inhibitors (SSRIs) other than fluoxetine: an 8-week open-label randomized clinical trial

**DOI:** 10.1186/s12888-024-05507-7

**Published:** 2024-01-23

**Authors:** Arman Alipour-Kivi, Negin Eissazade, Seyed Vahid Shariat, Razieh Salehian, Shiva Soraya, Sanaz Askari, Mohammadreza Shalbafan

**Affiliations:** 1 Mental Health Research Center, Psychosocial Health Research Institute (PHRI), Department of Psychiatry, School of Medicine, Iran University of Medical Sciences, Tehran, Iran; 2 Brain and Cognition Clinic, Institute for Cognitive Sciences Studies, Tehran, Iran; 3 Research Center for Addiction and Risky Behaviors (ReCARB), Psychosocial Health Research Institute, Department of Psychiatry, School of Medicine, Iran University of Medical Sciences, Tehran, Iran

**Keywords:** Selective serotonin reuptake inhibitors, Drug holidays, Sexual dysfunction, Erection, Satisfaction, Ejaculation

## Abstract

**Introduction:**

Selective serotonin reuptake inhibitors (SSRIs) are widely used for the treatment of various mental disorders. Sexual dysfunction is one of the most common side effects of SSRIs, and often leads to poor adherence and treatment discontinuation. While several strategies have been employed to manage SSRI-induced sexual dysfunction, drug holidays has not been extensively studied for this purpose. This clinical trial aims to assess the effect of drug holidays on sexual dysfunction in married men under treatment with SSRIs other than fluoxetine (as its long half-life makes drug holidays ineffective).

**Methods:**

This 8-week double-center, randomized, open-label, controlled trial was conducted in the outpatient clinics of Iran Psychiatric Hospital and Tehran Institute of Psychiatry, from January 2022 to March 2023. We included married men aged between18 and 50 years who had experienced sexual dysfunction during treatment with SSRIs, other than fluoxetine. The Male Sexual Health Questionnaire (MSHQ) and the 28-Question General Health Questionnaire (GHQ-28) were used for the assessment of sexual function and mental health status. The drug holidays group was instructed not to take their medications on the weekends. The control group was asked to continue their regular medication regimen without any changes. Both groups were assessed at baseline, and weeks 4 and 8.

**Results:**

Sixty-three patients were included and randomly assigned to the drug holidays group (*N* = 32) or the control group (*N* = 31). Fifty patients (25 in each group) completed the trial. Drug holidays significantly improved erection, ejaculation, satisfaction, and the overall sexual health of the participants (*P* < 0.001). No significant change was observed in their mental health status. No major side effects were recorded.

**Conclusions:**

Drug holidays significantly improved the MSHQ scores in ‘erection’, ‘ejaculation’, ‘satisfaction’ and ‘total’ in married men with sexual dysfunction induced by SSRIs, other than fluoxetine, without causing any significant changes in their mental health status. Further research is needed to reach a certain conclusion.

**Trial registration:**

The trial was registered at the Iranian Registry of Clinical Trials on 2021.10.25 (www.irct.ir; IRCT ID: IRCT20170123032145N6) before the trial.

## Background

Mental disorders are among the top ten leading causes of global burden of disease [1]. Selective serotonin reuptake inhibitors (SSRIs) are a cornerstone class of medications in psychopharmacology for treating conditions like depression, anxiety disorders, and obsessive-compulsive disorder. However, they are commonly associated with side effects that significantly impairdaily activities and lead to poor quality of life [2]. Sexual dysfunction is among the most common side effects of SSRIs, and it can lead to non-adherence and treatment discontinuation [3]. In rare cases, sexual dysfunction becomes permanent even after discontinuation of the medication, known as post-SSRI sexual dysfunction (PSSD) [4].

SSRIs can affect any of the sexual cycle phases and cause gender-specific sexual dysfunction. In women, SSRIs more commonly manifest as reduced libido, delayed orgasm, anorgasmia, and sexual arousal disorder. However, men often experience erectile dysfunction and delayed ejaculation [3, 5, 6].

The mechanism by which SSRIs cause sexual dysfunction is not yet fully understood. However, it is thought to be related to alterations in the level of serotonin, acetylcholine, noradrenaline, dopamine, nitric oxide, and prolactin [7]. Some of the medications that have been suggested to counteract this effect are sildenafil, tadalafil and vardenafil (phosphodiesterase-5 inhibitors), amantadine (dopamine and norepinephrine agonist), cyproheptadine (5-HT blocker), buspirone (5-HT1A receptor partial agonist), and bupropion (norepinephrine and dopamine agonist), mirtazapine (serotonin and norepinephrine agonist), modafinil (dopamine agonist), agomelatine (MT1 and MT2 receptors agonist and 5-HT2 receptors antagonist), yohimbine (alpha-2 blocker), bethanechol (acetylcholine agonist) and ginkgo biloba (herbal medication). However, combination strategies may cause unwanted and intolerable side effects [3, 5].

So far, the following strategies have been employed to manage SSRI-induced sexual dysfunction: the ‘wait-and-see’ strategy, behavior-changing techniques and psychotherapy, dose reduction, delaying the use of medication until after sexual activity, switching the antidepressant medication, adjuvant therapy, and the ‘drug holidays’ [3, 5, 8].

Drug holidays is defined as temporarily stopping or reducing the dose of medication. It has previously been used to help alleviate side effects or improve the effectiveness of the primary medication for the treatment of various mental disorders [9, 10].

Thus far, only one 4-week clinical trial (1995) has been conducted to assess the effect of drug holidays on the SSRI-induced sexual dysfunction, in which was reported that drug holidays improved sexual function in sertraline and paroxetine users, However, fluoxetine users did not experience any changes, which may be due to the long half-life of fluoxetine [11].

As SSRI-induced sexual dysfunction continues to be a major cause of discontinuation of treatment, and drug holidays has not been extensively studied for this purpose, and biological and hormonal factors impact the sexual function of men and women differently, we conducted this open-label clinical trial aiming to assess the effect of drug holidays on sexual dysfunction in men treated with SSRIs, other than fluoxetine, as the previous clinical trial reported no improvement in fluoxetine users.

## Methods

### Trial setting and design

This 8-week double-center, randomized, open-label, controlled trial was conducted in the outpatient clinics of Iran Psychiatric Hospital and Tehran Institute of Psychiatry (both affiliated with Iran University of Medical Sciences, Tehran, Iran) from January 2022 to March 2023.

### Participants

Participants were men aged between 18 and 50 years who had experienced sexual dysfunction during treatment with an SSRI, other than fluoxetine. As sexual intercourse is only legally accepted within the context of marriage in Iran, we only included married men in our study.

All patients were interviewed by a board-certified psychiatrist. Their medical records were reviewed. They were allin their maintenance course of treatment, with a stable condition over the past two months and no changes in medication dosage. The exclusion criteria were: (1) being under treatment with fluoxetine (as its long half-life makes drug holidays ineffective), (2) using medications with known sexual side effects (such as tricyclic antidepressants, typical antipsychotics, risperidone, biperiden and anticholinergics), and (3) poor medication adherence (reported by their treating psychiatrist).

Demographic data were recorded. The Male Sexual Health Questionnaire (MSHQ) was filled out at baseline and weeks 4 and 8 [12]. The 28-Question General Health Questionnaire (GHQ-28) was filled out at baseline and endpoint of the study to assess the possible changes in the mental health of participants [13]. In addition, the signs and symptoms of the adverse effects potentially associated with drug holidays were assessed using a structured checklist at each visit [14].

Participants were randomized using the block method (blocks of four) in two groups: the drug holidays group and the control group. The allocation sequence was concealed in sequentially numbered, opaque, sealed envelopes. The randomizer and statistical analyzer were separate individuals blinded to allocation.

### Instruments

We used a demographic questionnaire to record age, education level, employment status, medications, and past psychiatric history of participants.

The GHQ-28 consists of four subscales: somatic symptoms, anxiety and insomnia, social dysfunction, and depression. Each question is scored from zero to three. Lower scores indicate better state of health. The validity and reliability of the Persian version of GHQ-28 have been assessed in the study of Taqvai et al. [13, 15].

MSHQ is a self-administered questionnaire used to assess sexual function in men. It consists of 25 questions on erection (4 questions), ejaculation (8 questions), and satisfaction (13 questions) over the past month. It is scored on a five or six-point Likert scale. Higher scores indicate better sexual health. Fakhri et al. have measured the validity and reliability of the Persian version of MSHQ. Content validity index (CVI), content validity ratio (CVR), Spearman-Brown coefficient, and Cronbach’s alpha coefficient were reported as 0.9, 0.78, 0.79, and 0.84, respectively [12, 16].

### Interventions

The participants in the drug holidays group were asked not to take their medications on Thursdays and Fridays (as these days are the weekends in Iran and having sexual intercourse is more likely to occur) for eight weeks. Participants in the control group were asked not to make any changes in their treatment plans and use their medications as they were prescribed.

### Outcomes

The primary outcome measure was the between-group difference of MSHQ’s total and subscales scores from baseline to the eighth week. The secondary outcome measures were the between-group differences in GHQ-28 scores from baseline to the eighth week, and the frequency and severity of adverse effects.

### Sample size and statistical analysis

A between-group difference of four, a type I error of 5%, and a power of 80% were used, and a sample size of 50 (25 in each group) was calculated [11].

Continuous variables are presented as mean ± standard deviation. The continuous score variables were tested for sphericity by using Mauchly’s test. Repeated-measures ANOVA analysis and Friedman test were used to evaluate the effect of demographics and drug holidays with time on the MSHQ scores. Independent T-test and Mann-Whitney U‑test were used to compare the mean scores between the two groups at different time points. Mean differences were calculated with a confidence interval of 95%. A p-value of < 0.05 was considered statistically significant.

We also used linear mixed effect model analysis to the measure the independent effect of drug holidays on MSHQ scores. A separate analysis was performed for the scores of each MSHQ subscale. The primary model included the mean score of each subscale as the dependent variable, and group, medication, education level, employment status, and time, as well as interactions of group-by-time and medication-by-time as coefficients of the fixed effect model, time as a coefficient for the random effect model, and the baseline scores, age, and GHQ scores as covariates. The control group and the third time point were used as index groups for comparison, with their values set to zero. Medication, education level, employment status, and medication-by-time interaction did not show a significant effect and were excluded from the final models.

All statistical analyses were performed with the Statistical Package for the Social Sciences (SPSS) software for Windows (version 27, SPSS Inc., Chicago, IL, USA).

## Results

### Participants

**Fig. 1:**
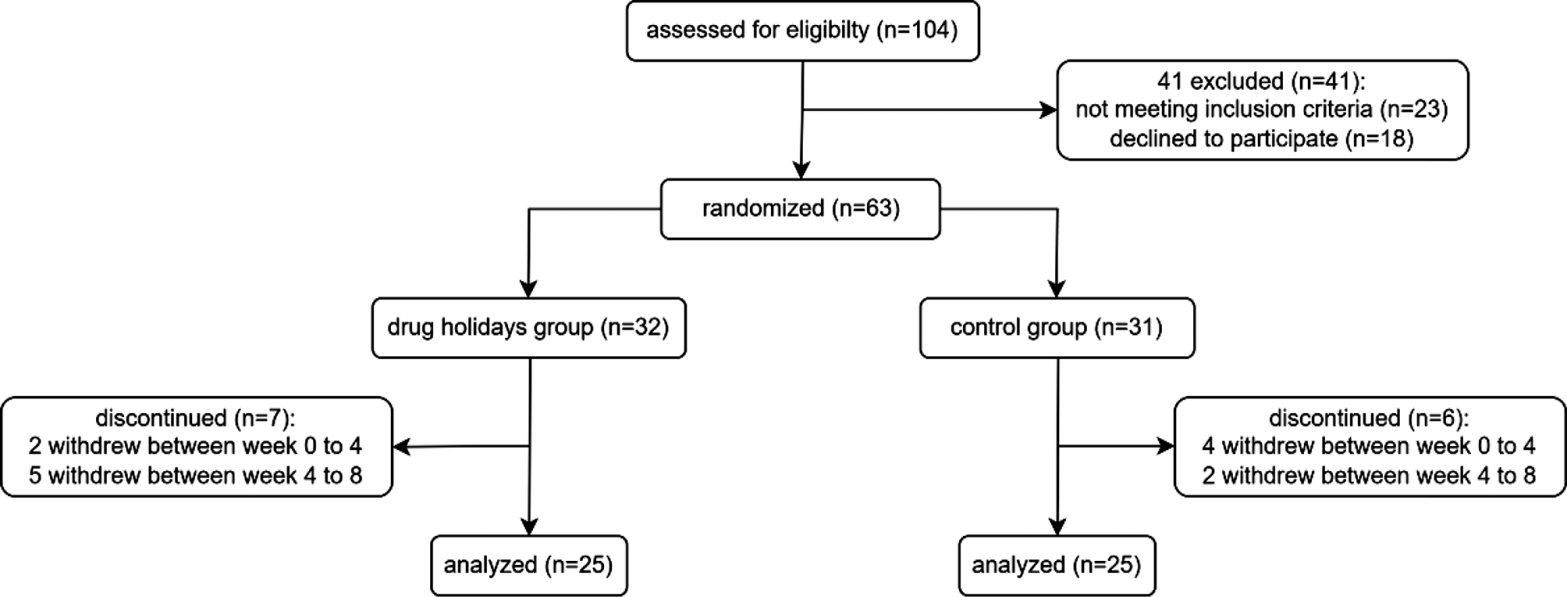
Flow diagram of the participants of the trial

Sixty-three patients were included in the study and randomly assigned to the drug holidays group (*N* = 32) or the control group (*N* = 31). Fifty patients (25 in each group) completed the trial. The participants’ mean (± SD) age was 37.22 (± 12.181) years. The demographic characteristics of the participants are presented in Table 1. The flow diagram of the participants is presented in Fig. 1.

**Table 1:** Demographic data of the participants

		Drug holidays group (*N* = 25)			Control group (*N* = 25)		
		Mean (± SD)	Count (%)		Mean (± SD)	Count (%)	
Age (years)		36.44 (**±** 6.049)			35.04 (**±** 6.693)		
Education level	Illiterate		-			1 (4%)	
	High school diploma or lower		5 (20%)			10 (40%)	
	Higher education		20 (80%)			14 (56%)	
Employment status	Employed		20 (80%)			19 (86%)	
	Unemployed		5 (20%)			6 (24%)	
Medication	Sertraline		11 (44%)			9 (36%)	
	Escitalopram		7 (28%)			9 (36%)	
	Paroxetine		1 (4%)			1 (4%)	
	Citalopram		4 (16%)			4 (16%)	
	Fluvoxamine		2 (8%)			2 (8%)	
Previous psychiatric diagnosis	Depressive disorders		13 (52%)			6 (24%)	
	Anxiety disorders		10 (40%)			12 (48%)	
	Obsessive-compulsive and related disorders		2 (8%)			7 (28%)	

The baseline mean scores of MSHQ were not significantly different between the two groups (Table 2).

**Table 2:** MSHQ scores of the participants in total, and the erection, ejaculation and satisfaction subscales (mean ± standard deviation) and the mean difference between the groups at baseline and weeks 4 and 8

		Drug holidays group (*N* = 25)	Control group (*N* = 25)	P-value
Erection	Baseline	10.96 ± 2.865	10.04 ± 2.541	0.23
	Week 4	11.80 ± 2.598	9.08 ± 2.768	0.001*
	Week 8	12.52 ± 2.044	8.72 ± 2.807	< 0.001*
Ejaculation	Baseline	25.20 ± 5.612	26.60 ± 5.958	0.39
	Week 4	26.80 ± 5.881	25.60 ± 6.357	0.49
	Week 8	28.32 ± 5.336	26.08 ± 6.041	0.171
Satisfaction	Baseline	21.08 ± 5.220	19.24 ± 5.995	0.26
	Week 4	23.00 ± 4.435	17.80 ± 6.813	0.005*
	Week 8	25.72 ± 4.078	18.28 ± 6.580	< 0.001*
Total	Baseline	57.24 ± 11.780	55.88 ± 12.022	0.68
	Week 4	60.88 ± 11.114	52.52 ± 13.194	0.019*
	Week 8	66.56 ± 9.820	53.12 ± 12.982	< 0.001*

*P-values less than 0.05

### MSHQ total scores

**Fig. 2:**
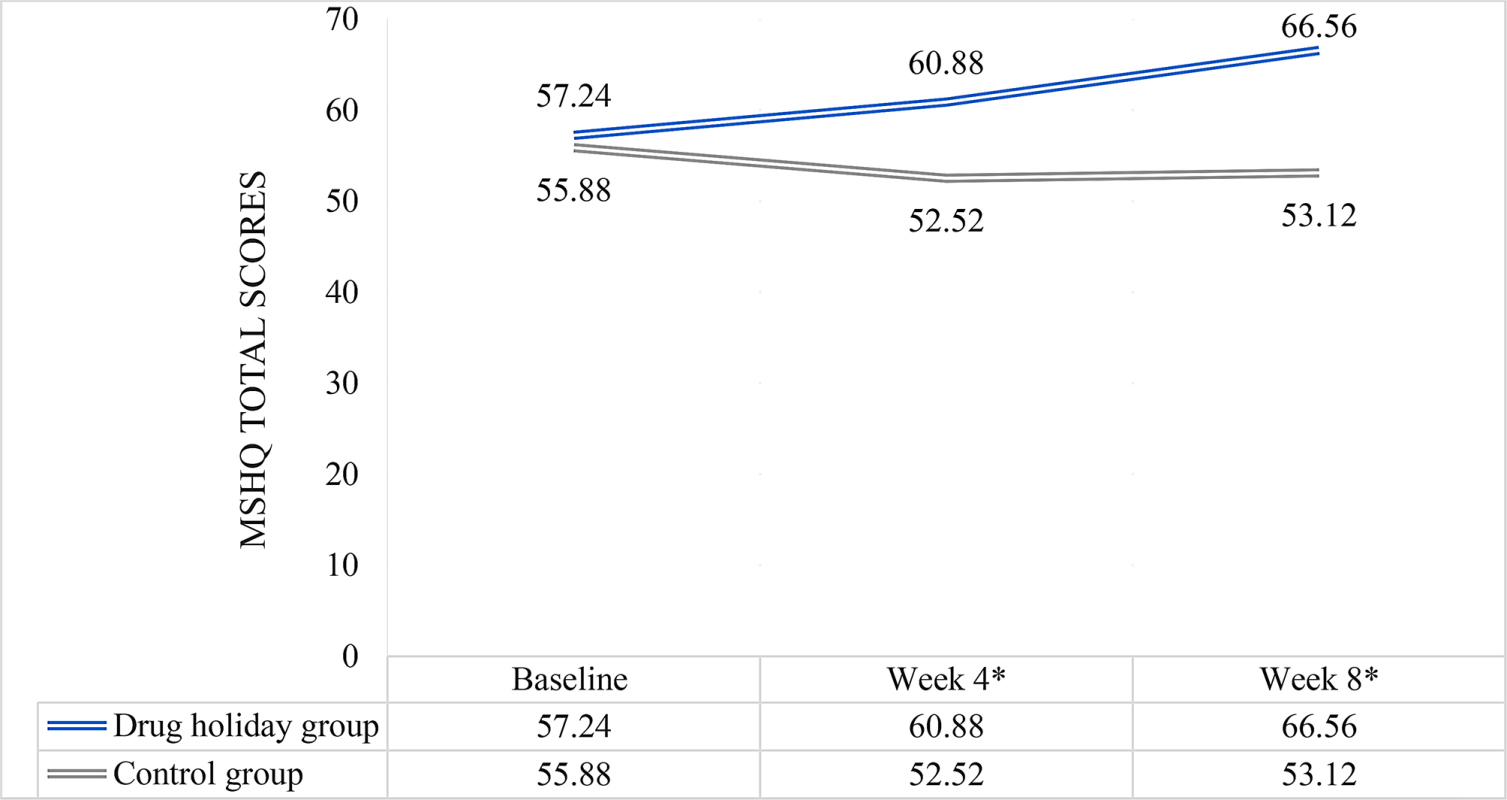
MSHQ total score changes of the participants during the course of trial. *significant difference between the two groups

The mean total score increased from 57.24 ± 11.780 at baseline to 66.56 ± 9.820 at the end of trial in the drug holidays group, and decreased from 55.88 ± 12.022 to 53.12 ± 12.982 in the control group (Fig. 2). Comparison of the means revealed significant difference between the groups at weeks 4 (*P* = 0.019) and 8 (*P* < 0.001) (Table 2).

In total, the mean score changes between the groups, were statistically significant (Huyn-Feldt F(1.774, 85.174) = 9.44, *P* < 0.001). Repeated-measures ANOVA analysis detected a significant Time X Treatment interaction in both drug holidays group, (F(2, 48) = 24.60, *P* < 0.001), and the control group (Huynh-Feldt F(1.653, 39.663) = 5.728, *P* = 0.010).

Linear mixed effect model analysis revealed that the effect of time (*P* < 0.001), group (*P* < 0.001), baseline scores (*P* < 0.001) and Group * Time interaction (*P* < 0.001) was significant. Drug holidays group had a significantly higher total score, regardless of time of assessment (*P* < 0.001). Moreover, total score was not significantly different from baseline at neither week 4 (*P* = 0.620) nor week 8 (*P* = 0.024) among the drug holidays group. However, the Group * Time interaction was significant at both weeks 4 (*P* = 0.004) and 8 (*P* = 0 < 0.001). Random effect analysis revealed an unmeasured confounding factor (*P* = 0.006).

### MSHQ erection scores

**Fig. 3:**
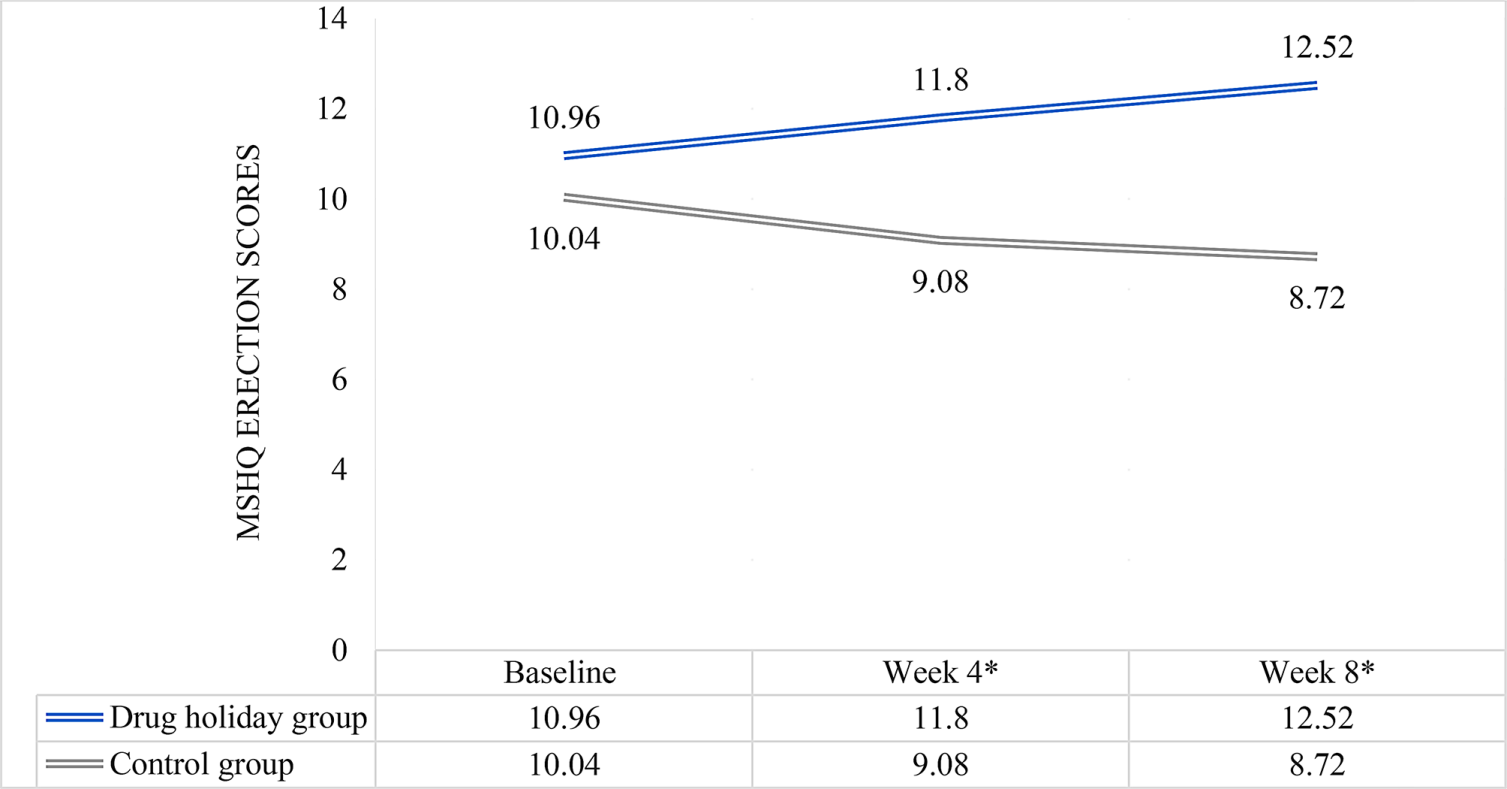
MSHQ erection score changes of the participants during the course of trial. *significant difference between the two groups

The mean satisfaction score increased from 10.96 ± 2.865 to 12.52 ± 2.044 in the drug holidays group, and decreased from 10.04 ± 2.541 to 8.72 ± 2.807 in the control group (Fig. 3) (Table 2). Comparison of the means revealed significant difference between the groups at weeks 4 (*P* = 0.001) and 8 (*P* < 0.001) (Table 2).

Repeated-measures analysis revealed that the mean erection score change was statistically significant between the groups (Huyn-Feldt F(1.354, 32.505) = 4.899, *P* = 0.024). In addition, the mean score change was significant among the drug holidays group (F(2, 48) = 10.134, *P* < 0.001), and non-significant among the control group (Greenhouse-Geisser F(1.309, 31.422) = 4.899, *P* = 0.26).

In the linear mixed effect model analysis, the following variables showed a significant effect for erection: group (*P* < 0.001), baseline erection score (*P* < 0.001) and Group * Time interaction (*P* < 0.001); however, time effect by itself was not significant (*P* = 0.8). Drug holidays group had a significantly higher score of erection, regardless of time of assessment (*P* < 0.001). Moreover, erection score was significantly higher at week 8 (*P* < 0.001) among the drug holidays group, but not at week 4 (*P* = 0.363). Also, after controlling for the baseline score and using the control group as an index for comparison, Group * Time interaction was significant at week 4 (*P* = 0.056), but only marginally, as well as at week 8 (*P* < 0.001), indicating that erectile function gradually improved over the course of treatment. However, random effect analysis revealed the effect of an unmeasured confounding factor (*P* = 0.01).

### MSHQ ejaculation scores

**Fig. 4:**
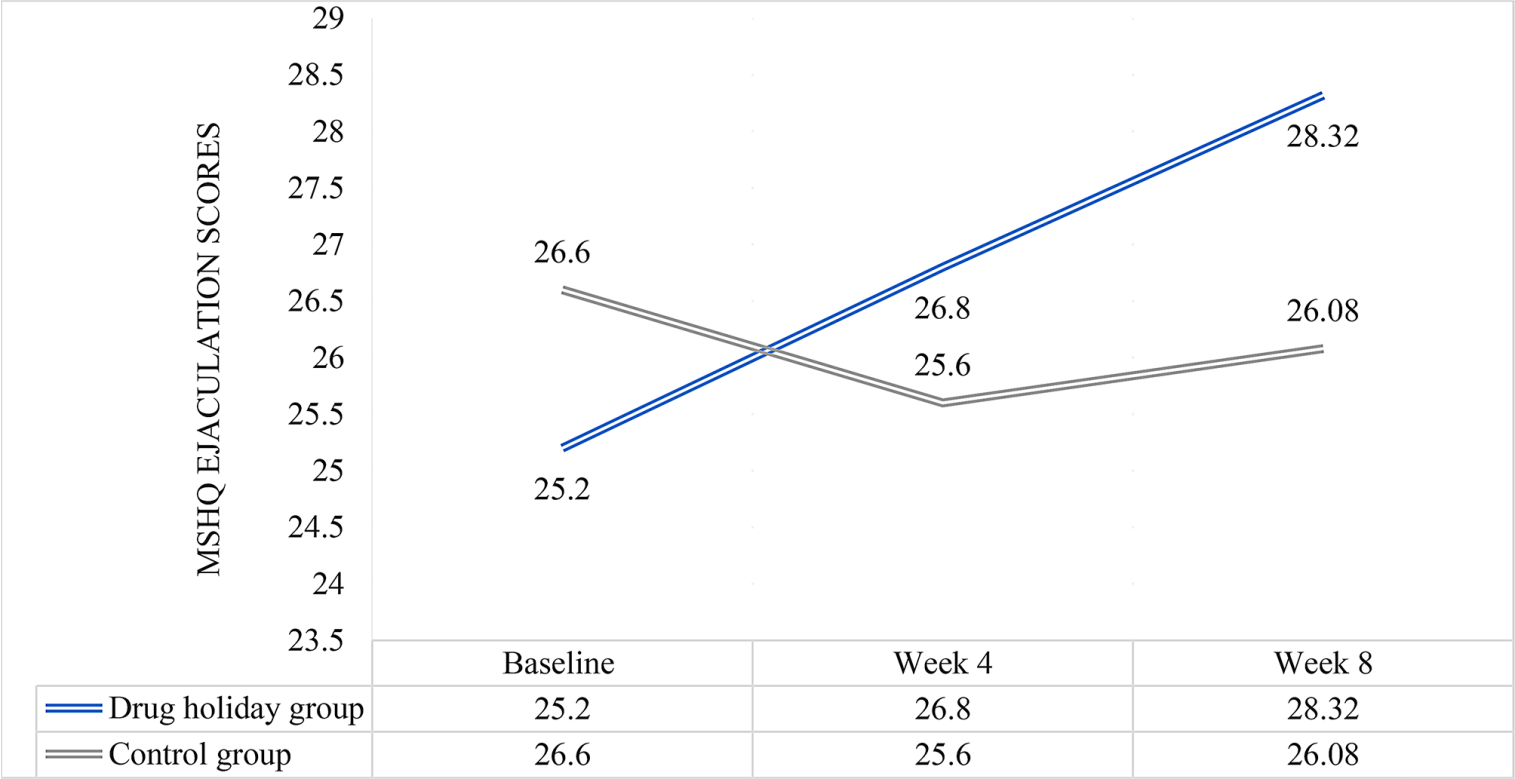
MSHQ ejaculation score changes of the participants during the course of trial

The mean of satisfaction scores increased from 25.20 ± 5.612 to 28.32 ± 5.336 in the drug holidays group, and slightly decreased from 26.60 ± 5.958 to 26.08 ± 6.041 in the control group (Fig. 4) (Table 2). Unlike comparison of the means by Mann-Whitney U‑test that did not reveal any significant difference (Table 2), repeated-measures ANOVA detected that mean score change was statistically significant between the groups, F(2, 96) = 17.494, *P* < 0.001. Additionally, the Time X Interaction effect was significant among the drug holidays group (F (2, 48) = 26.484, *P* < 0.001), and non-significant among the control group (F(2, 48) = 2.294, *P* = 0.112).

As revealed by the linear mixed effect model analysis, the following variables had a significant effect on ejaculation: time (*P* < 0.001), group (*P* < 0.001), baseline erection score (*P* < 0.001) and Group * Time interaction (*P* < 0.001). Drug holidays group had a significantly higher score of ejaculation, regardless of time of assessment (*P* < 0.001). Ejaculation score was not significantly different at neither week 4 (*P* = 0.287) nor week 8 (*P* = 0.249) among the drug holidays group. However, the Group * Time interaction was non-significant at week 4 (*P* = 0.104) and significant at week 8 (*P* < 0.001). Random effect analysis revealed the effect of an unmeasured confounding factor (*P* = 0.006).

### MSHQ satisfaction scores

**Fig. 5:**
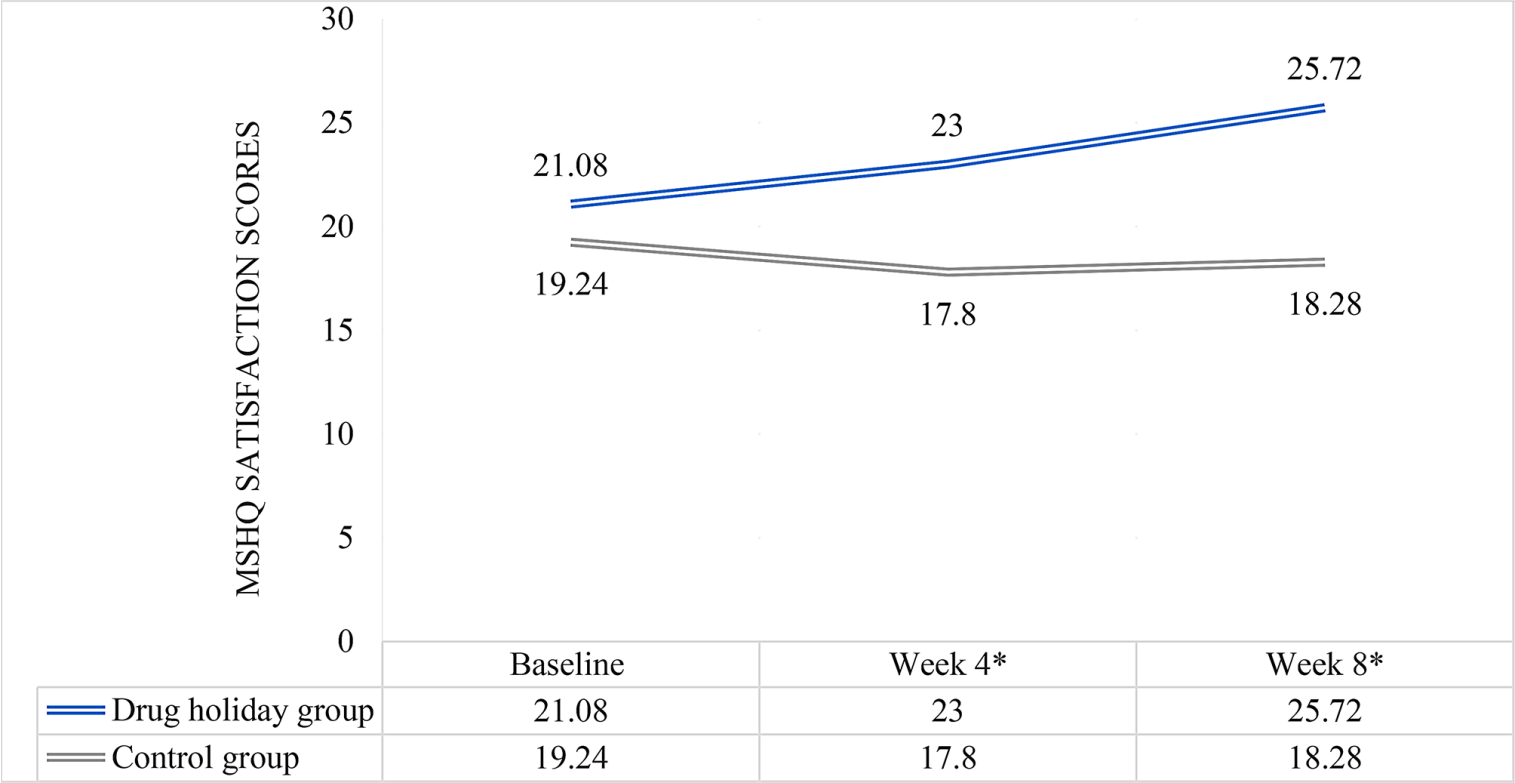
MSHQ satisfaction score changes of the participants during the course of trial. *significant difference between the two groups

The mean of satisfaction scores increased from 21.08 ± 5.220 to 25.72 ± 4.078 in the drug holidays group, and decreased from 19.24 ± 5.995 to 18.28 ± 6.580 in the control group (Fig. 5) (Table 2). Comparison of the means revealed significant difference between the groups at weeks 4 (*P* = 0.005) and 8 (*P* < 0.001) (Table 2).

Friedman test’s revealed a significant change in the mean satisfaction score in both drug holidays (χ^2^(2, *N* = 25) = 28.295, *P* < 0.001) and control (χ^2^(2, *N* = 25) = 6.997, *P* = 0.31) groups during the course of trial. Friedman test’s mean rank scores are presented in Table 3.

**Table 3:** Friedman test’s mean rank scores for the satisfaction subscale of MSHQ

	Drug holidays group (N = 25)	Control group (N = 25)
Baseline	1.36	2.36
Week 4	1.84	1.66
Week 8	2.8	1.98

For satisfaction, the following variables showed a significant effect by linear mixed effect model analysis: time (*P* < 0.001), group (*P* < 0.001), baseline score (*P* < 0.001) and Group * Time interaction (*P* < 0.001). Drug holidays group had a significantly higher score of satisfaction, regardless of time of assessment (*P* < 0.001). Moreover, satisfaction score was not significantly different from baseline at neither week 4 (*P* = 0.462) nor week 8 (*P* = 0.143) among the drug holidays group. However, the Group * Time interaction was significant at both weeks 4 (*P* = 0.017) and 8 (*P* < 0.001). Random effect revealed an unmeasured confounding factor (*P* = 0.006).

### GHQ-28 scores

GHQ-28 scores decreased in both groups, indicating improvement of mental health, which was not significant in the drug holidays group (*P* = 0.066).

### Side effects

Patients in the drug holidays group reported experiencing nausea (16%, *N* = 4), headache (24%, *N* = 6) and mild restlessness (24%, *N* = 6). None of the patients in the control group reported any additional side effects.

## Discussion

Based on the results of our study, drug holidays was significantly in favor of ‘erection’, ‘satisfaction’, ‘ejaculation’ and ‘total’ scores of the MSHQ, indicating improvement of sexual health.

Thus far, only one clinical trial conducted by Rothschild et al. (1995) has investigated the effect of the drug holidays on the sexual dysfunction induced by SSRIs. Compared to our study, they had a shorter period (four weeks) and a smaller sample size (14 men). They recruited 14 men and 16 women under treatment with sertraline, paroxetine, and fluoxetine. None of the patients took high doses of SSRIs, so the withdrawal symptoms were not likely to appear. Patients were asked not to take their medications after the Thursday morning dose until Sunday noon for four weeks. Male patients who were taking sertraline and paroxetine reported improved orgasm function (60%), sexual satisfaction (50%), and libido (50%) without a significant increase in mean Hamilton depression score. However, fluoxetine users did not report any improvements, which may be due to the long half-life of fluoxetine. Similarly, we found improvement in erection, satisfaction, and overall sexual health, without significant worsening of the mental health status. Although, we did not assess libido [11].

The exact mechanism of how SSRIs cause sexual dysfunction is not yet clear. The proposed contributing mechanisms are as follows: serotonin receptor down-regulation, decreased levels of dopamine and norepinephrine, up-regulation of prolactin (leads to increased levels of sexual hormone-binding globulin (SHBG) and reduced levels of free testosterone), disruption of oxytocin signaling (reduced blood flow to genitals), altered activity of hypothalamic-pituitary-gonadal axis (decreased levels of testosterone and estrogen), disruption of nitric oxide pathway (reduced blood flow to genitals), and impacting the autonomic nervous system (e.g., genital numbness). Additionally, this process can be complicated by genetic variations (e.g., CYP2C19 and CYP3A4) alternating the metabolism of SSRIs and comorbid psychiatric disorders contributing to sexual dysfunction [17–23].

A Cochrane review (2013) was conducted on the management strategies or SSRI-induced sexual dysfunction. Other than drug holidays, the ‘wait-and-see’ strategy, behavior-changing techniques and psychotherapy, dose reduction, delaying the use of medication until after sexual activity, switching to a different antidepressant, and adjuvant therapy have been proposed. Most interventions have not been studied in clinical trials. Limited evidence, potential side effects, and variable mechanisms of action make it challenging to make a conclusive comparison [3, 8].

The ‘wait-and-see’ strategy, ‘behavior-changing techniques’, and ‘psychotherapy’ may lead to a gradual resolution of sexual dysfunction. However, they usually require a significant amount of time and commitment and may lead to prolonged dissatisfaction. ‘Dose reduction’ and ‘delayed doses of medication until after sexual activity’, can potentially disrupt the stability of mental health treatment and lead to a recurrence of symptoms or withdrawal symptoms. Moreover, they may require close monitoring by healthcare professionals. ‘Switching to a different antidepressant’ can involve a period of adjustment, and the new medication may still have potential side effects. ‘Adjuvant therapy’ can also introduce new potential side effects. For instance, sildenafil, tadalafil, and vardenafil (phosphodiesterase 5 inhibitors) have a rapid onset of action, but they can commonly cause headache, flushing, and dyspepsia. Buspirone (5-HT1A receptor partial agonist) can cause dizziness, nausea, and headaches. Bupropion (norepinephrine and dopamine agonist) can cause insomnia, agitation, and increased heart rate [3–9, 24].

Compared to the previous strategies, drug holidays is simple and it can potentially improve treatment adherence and reduce the likelihood of treatment discontinuation due to sexual dysfunction, by providing a temporary respite from the side effects. It has been recommended to be used for orgasm delay or anorgasmia in women. Notably, the costs and benefits of the drug holiday should be weighed for each patient. The decision to implement this method should be made in collaboration between the patient and their healthcare provider, taking into account the specific characteristics of the mental disorder, the severity of sexual dysfunction, and the potential risks and benefits of temporary discontinuation of the medication [6, 21]. Our study provided evidence for the positive effect of drug holidays on erection, satisfaction, and overall, sexual health in men. However, further research is needed to determine the safety and efficacy of this method.

### Limitations

Our study was limited by a small sample size, short follow-up period, self-report bias, inclusion of only married men (selection bias), exclusion of patients with comorbidities, the use of different SSRIs among patients (mostly sertraline and escitalopram), and the use of different doses of SSRIs among the patients, which limited generalizability of our results. Multi-center clinical trials with extended follow-up periods and large sample sizes are needed to shape the body of evidence for the safety and efficacy of drug holidays.

## Conclusions

Based on the results of our study, employing drug holidays for the treatment of SSRI-induced (except fluoxetine) sexual dysfunction in married men significantly improved ‘erection,’ ‘ejaculation,’ ‘satisfaction,’ and ‘total’ scores of the MSHQ. Further multi-center clinical trials with extended follow-up periods and larger sample sizes are needed to reach a certain conclusion.

## Data Availability

The datasets generated and/or analysed during the current study are not publicly available due to confidentiality concerns (in the informed consent, we have made a commitment to the participants to publish only the general and group results of the study) but are available from the corresponding author on reasonable request.

## Abbreviations

SSRI: Selective Serotonin Reuptake Inhibitor
MSHQ: Male Sexual Health Questionnaire
GHQ-28: 28-Question General Health Questionnaire
CVI: Content validity index
CVR: content validity ratio
SPSS: Statistical Package for the Social Sciences
SHBG: sexual hormone-binding globulin
